# Contemporaneous Trace and Body Fossils from a Late Pleistocene Lakebed in Victoria, Australia, Allow Assessment of Bias in the Fossil Record

**DOI:** 10.1371/journal.pone.0052957

**Published:** 2013-01-02

**Authors:** Aaron Bruce Camens, Stephen Paul Carey

**Affiliations:** 1 Biological Sciences, Flinders University, Bedford Park, Australia; 2 Centre for Environmental Management, School of Science, Information Technology and Engineering, University of Ballarat, Ballarat, Australia; Ludwig-Maximilians-Universität München, Germany

## Abstract

The co-occurrence of vertebrate trace and body fossils within a single geological formation is rare and the probability of these parallel records being contemporaneous (i.e. on or near the same bedding plane) is extremely low. We report here a late Pleistocene locality from the Victorian Volcanic Plains in south-eastern Australia in which demonstrably contemporaneous, but independently accumulated vertebrate trace and body fossils occur. Bite marks from a variety of taxa are also present on the bones. This site provides a unique opportunity to examine the biases of these divergent fossil records (skeletal, footprints and bite marks) that sampled a single fauna. The skeletal record produced the most complete fauna, with the footprint record indicating a markedly different faunal composition with less diversity and the feeding traces suggesting the presence, amongst others, of a predator not represented by either the skeletal or footprint records. We found that the large extinct marsupial predator *Thylacoleo* was the only taxon apparently represented by all three records, suggesting that the behavioral characteristics of large carnivores may increase the likelihood of their presence being detected within a fossil fauna. In contrast, *Diprotodon* (the largest-ever marsupial) was represented only by trace fossils at this site and was absent from the site's skeletal record, despite its being a common and easily detected presence in late Pleistocene skeletal fossil faunas elsewhere in Australia. Small mammals absent from the footprint record for the site were represented by skeletal fossils and bite marks on bones.

## Introduction

Trace fossils can provide both behavioral and morphological information about organisms that is not preserved in the body (skeletal) fossil record. In addition, trace fossils are a source of important information about intraspecific and interspecific faunal interactions. In some cases trace fossils can also indicate an extension of the temporal or spatial range of a taxon beyond that known from skeletal fossils (e.g. [1], [2], [3], [4], [5]). It is rare that a vertebrate trace fossil can be definitively allocated to a species described from skeletal material [5], [6] and even rarer that vertebrate trace and body fossils occur together [5].

Examination of faunal composition and diversity in paleocommunities is integral to understanding both past and present ecosystems. It has long been acknowledged that fossil assemblages provide a generally incomplete record of paleodiversity and relative species abundance due to taphonomic biases (e.g. [7]). Paleontologists thus often rely on the presence of particular species combinations (e.g. [8], [9]) or key indicator species (e.g. [7]) when forming paleoecological or paleoenvironmental hypotheses. Lockley [10] observed that fossil tracks are “much more likely to represent a valid census of a living community than remains found at the majority of skeletal sites”. This is because fossil footprints are not subject to the same degree of time-averaging as body fossils [5], [10], and so paleoecological information concerning species interactions derived from trace fossils is likely to be more accurate than that derived from body fossils.

### Co-occurrence of trace and body fossils

The rarity of documented Pleistocene vertebrate trace fossils, as compared to body fossils, has been noted both within Australia [11], [12] and worldwide [13]. It has also been observed that the paleoecological investigation of dinosaurian ichnocoenoses (trace fossils from a number of taxa recorded in a single horizon [14]) is uncommon [2], [10]. Recent or modern mammalian ichnocoenoses have received some attention [15], [16], [17], [18] but, until now, comparison of a terrestrial vertebrate ichnocoenosis with a penecontemporaneous skeletal fossil fauna from the same location has not been possible.

Near-contemporaneous dinosaur bones and trackways have been reported from the Upper Cretaceous Dunvegan Formation of British Columbia [19], [20]. Both trace and body fossils have also been found in the Upper Jurassic Morrison Formation of Colorado (dinosaurian and pterosaurian: [21], [22]), the Upper Jurassic–Lower Cretaceous Kuwajima and Kitadani Formations of Japan [23] and the Joggins Formation of Nova Scotia (tetrapod) [24]. Mammal trace and body fossils have been reported from the late Miocene Namurungule Formation of Kenya [25], [26], [27], the Miocene Toro Negro Formation of Argentina [28], the Miocene Siwalik Group [29], the Plio-Pleistocene Koobi Fora Formation of Kenya [30], the late Quaternary lower Wentlooge Formation of the Severn Estuary, UK [16], late Pleistocene sediments in southwestern Alberta, Canada [13], [31], and 12–16 ka sediments in Buenos Aires Province of Argentina [32]. Several authors have also discussed sites in which trampling has forced the burial of skeletal elements (e.g. [10], [30], [33]). In most of these cases the formation of the trace fossil and the skeletal fossil deposits is separated by a significant period of time and, in the few cases where the two records appear near-contemporaneous, discussion of the faunas represented by the two records has been limited to spatiotemporal considerations. Matsukawa et al. [23] concluded that dinosaurian skeletal faunas in East Asia could not be easily compared to trackway assemblages due to the large-scale time averaging of the former. In contrast to the above records, the relatively short depositional timeframe (tens of years) of the skeletal deposits at the Victorian Volcanic Plains (VVP) site described below (an interval that also included the deposition of the footprints at the site) enables a rare and valuable comparison of the inherent biases of several types of fossil record to be made.

Here we explore the respective biases of the ichnological and skeletal records of vertebrate activity at a late Pleistocene locality in the volcanic plains of Victoria, Australia. Late Pleistocene skeletal fossils are known from several locations in the region including Lakes Weering, Corangamite, Colongulac and Weeranganuk [34], [35], [36] allowing contrast between the faunal record represented at this site and those already published. The trace fossils at the VVP site include both footprint and feeding (bite marks and digestive etching) types. Our intent is twofold: (1) to demonstrate how contemporaneous trace and body fossils can contribute to a more complete assessment of the biodiversity of ancient ecosystems than either usually does on its own, and (2) to demonstrate the limitations of each type of fossil preservation.

## Results

### Geological setting

The skeletal accumulations and the trackways at the site are part of a volcaniclastic sandstone (>0.5 m thick) which underlies a partially eroded dolomitic limestone (100–150 mm thick). Both are part of an informal unit of lacustrine and associated aeolian deposits located in the VVP, which overlies the Newer Volcanic Group [37]. Although the dolomitic limestone is currently being stripped by modern erosional processes, its presence is a factor in the preservation of both the trackways and the skeletal accumulations. The upper surface, on which the trackways are preserved [12], is organised into sand bars of wavelength 10–15 m and amplitude 10–15 cm which trend north-south and prograded eastward. Carey et al. [12] noted that a small component of smectite in the sandstone was probably responsible for the fine moulding of the trackways, and that the impregnation with calcitic cement of the uppermost 10 mm of most of the trackway surface was critical to the preservation of the trackways.

**Figure 1:**
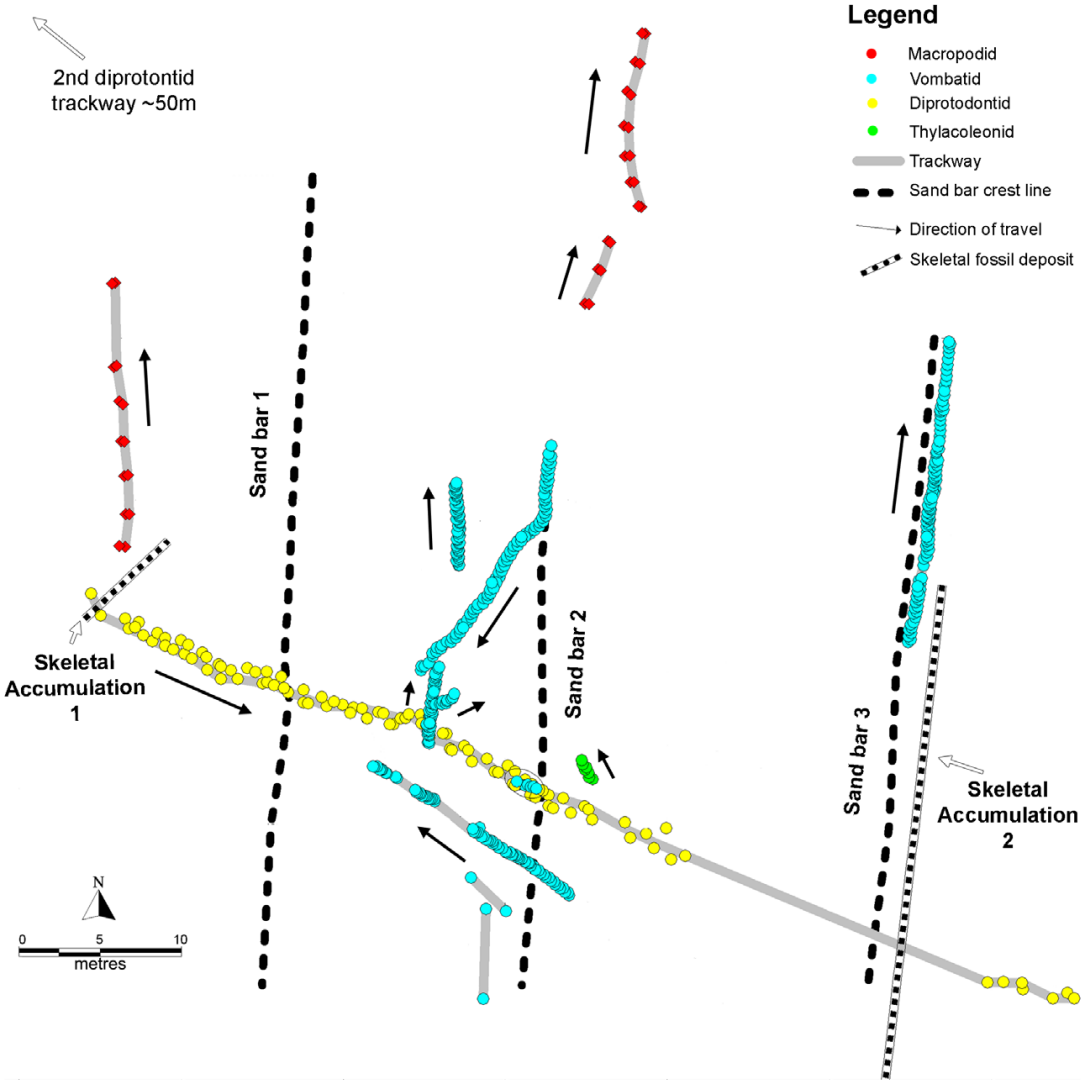
Map of the VVP site. Plan view of the distribution of the two skeletal deposits in relation to the trackways at the Victorian Volcanic Plains site.

The two skeletal accumulations (see Table 1 for dimensions) lie within the uppermost part of the volcaniclastic sandstone. At both skeletal sites the upper sediment is an unconsolidated sandy mud containing easily extracted fossil bones and teeth. The lower sediments of each site are cemented, usually with iron oxyhydroxide, to form volcaniclastic sandstone, with skeletal fossils embedded. Skeletal Accumulation 1 (SA1) transects the proximal end of a *Diprotodon* trackway, and is elongate in a NE–SW direction (see Figure 1). Despite its trench-like character at the surface, it lacks a distinct, channelized base. Instead, its lower part is continuous with the volcaniclastic host of the diprotodontid trackway. Skeletal Accumulation 2 (SA2) is at the toe of the east-dipping slope of one of the sand bars, just below part of a vombatid trackway that extends along the sand bar's slope (see Figure 1). SA2 is elongate in a N-S direction, and appears to have formed in the trough adjacent to the sand bar. As the skeletal deposits occur stratigraphically below and above the cemented trackway bearing surface, it is clear that they were formed at approximately the same time as the footprints, rather than being reworked from an older or younger layer.

**Table 1:** Body fossil deposit dimensions.

	SA 1	SA 2
length	7.0	33.0
width	0.5	0.8
depth	0.3	0.3

Approximate dimensions of skeletal accumulations (SA) in metres. For location relative to trackways, see Figure 1.

### Geochronology

Carey et al. [12] used a variety of dating techniques to estimate the age of the VVP trackways deposit. The analyses included OSL dating of the matrix in which the bones of SA2 were preserved, yielding a minimum age of 57 ka, and OSL dating of the volcaniclastic host to the trackways, yielding a minimum age of 75 ka. Combined U-series/ESR dating of teeth from SA2 yielded a best estimate of 98±15 ka. U-Th dating of the dolomitic limestone overlying the volcaniclastic sediments bearing the trace and body fossils gave a minimum age of 60±7 ka for secondary calcite accumulation within it. Together, the various ages suggest that the skeletal accumulations formed at some time in the interval, 60–110 ka.

**Figure 2:**
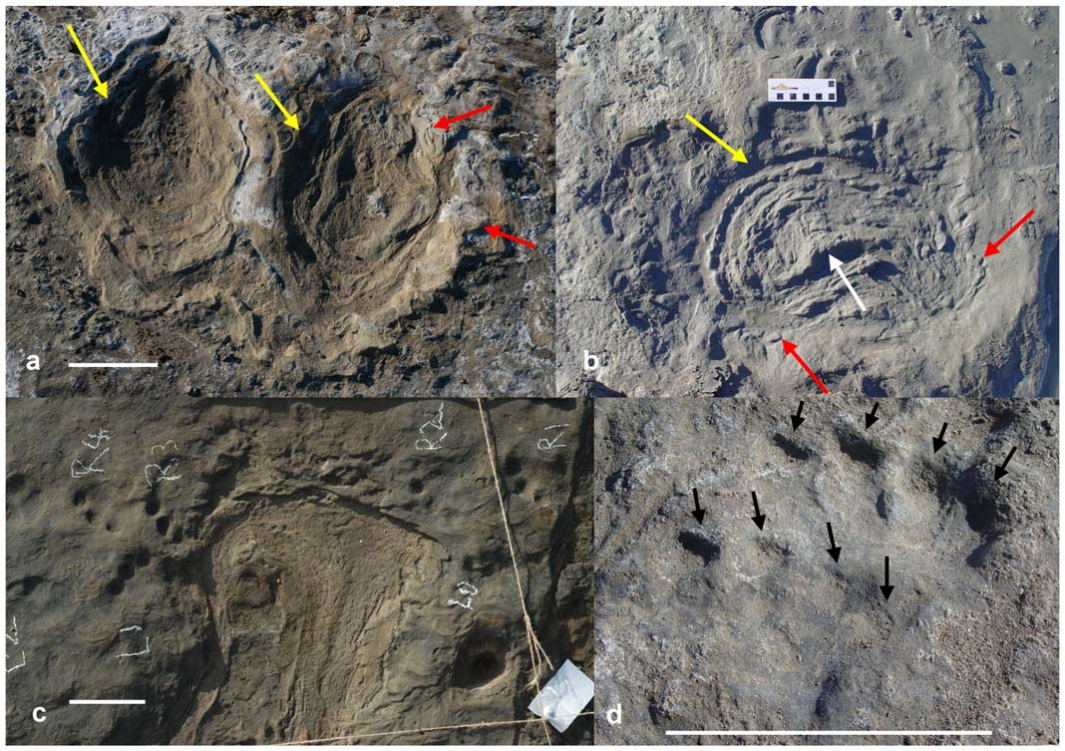
Fossil footprints from the VVP site. (a) large macropodid tracks, identified in [12] as probably belonging to the extinct macropodid *Protemnodon*, illustrating deformational characteristics that give clues as to the paleoenvironment of the trackway surface; (b) diprotodontid pes print, identified in [12] as belonging to *Diprotodon*; (c) a diprotodontid pes print overprinting a vombatid trackway; (d) possible *Thylacoleo* prints, black arrows point to digital impressions; all scale bars equal 100 mm, yellow arrows point to marginal ridges, red arrows to ejecta and white arrows to adhesion ridges.

Careful stratigraphic examination of the site by Carey et al. [12] revealed that, with one or two possible exceptions, the trackways were most likely restricted to a single bedding surface. The presence of features such as ejecta, marginal ridges and adhesion ridges in the diprotodontid and macropodid trackways (Figure 2) rules out the possibility of the tracks being underprints, and foot-pad detail in the tracks of the smaller quadrupeds also suggests a short period of accumulation of the footprints. The geochronology and stratigraphic position of SA2, in the upper layers of the volcaniclastics and below the (now-eroded) dolomitic limestone, indicate that the trackways situated on the surface of the volcaniclastics were imprinted during the period in which SA2 was deposited. While not conclusive, the suite of age estimates allows for virtually immediate burial of the trackways surface, SA1 and SA2 by the dolomitic limestone.

Importantly, the accumulation of skeletal remains occurred independent of the formation of the fossil footprints (i.e. bones were not trapped in the depressions created by the footprints). This contrasts with sites where bioturbation has been directly responsible for skeletal preservation (e.g. [30]), and allows comparison of two different types of fossil record produced from the same biocoenosis.

### Skeletal deposit sedimentology/taphonomy

**Figure 3:**
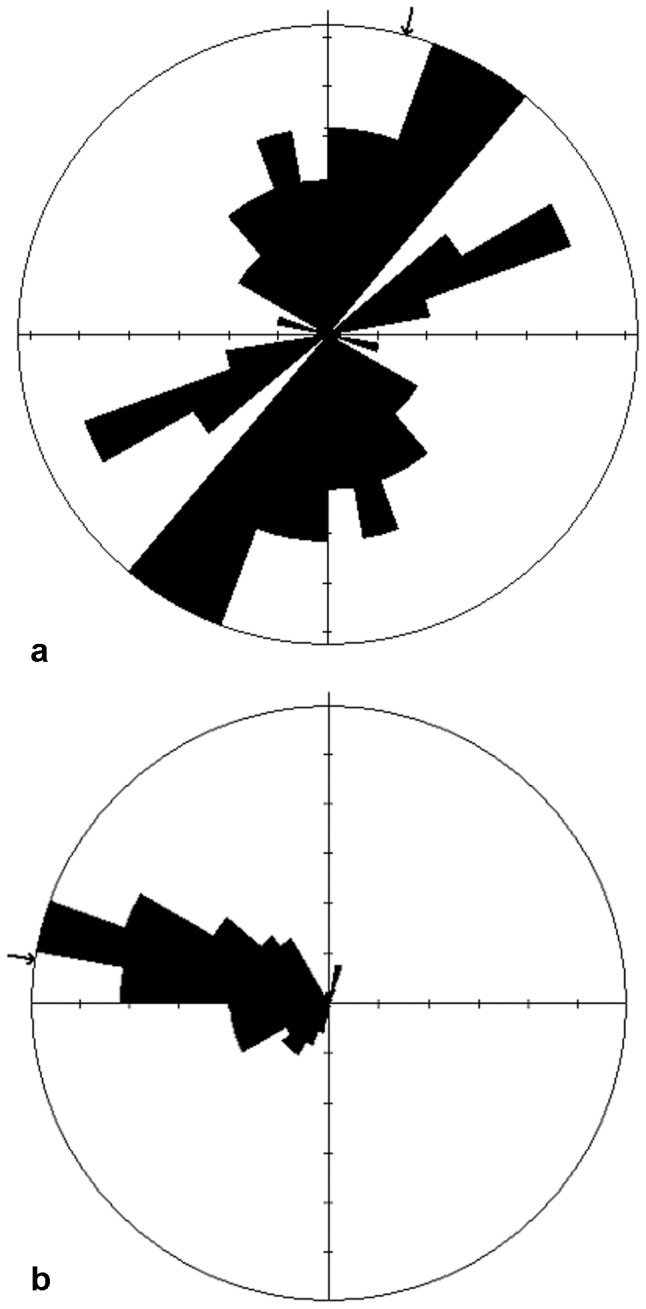
Ripple and bone orientation at the VVP site. (a) Rose diagram showing generally NNE-SSW alignment of elongate bones from skeletal accumulation 2. The mean (arrowed) of 48 measurements is 14° (95% confidence interval ±35°). (b) Rose diagram showing generally westward transport direction of straight-crested and linguoid ripples. The mean transport direction (arrow) is to 278° (95% confidence interval ±4°). The steeper face of each ripple is the lee slope. In the small proportion of ripples that are symmetrical, it is assumed that they were formed by waves travelling westward (onshore). Software used is Holcombe's GEOrient.

The large number of limb elements in SA2 permitted an analysis of bone orientation (Figure 3) that revealed the general NNE-SSW alignment of the bones, close to parallel with the adjacent N-S sand bar. The bones were also oriented parallel to the predominant ripple orientation (Figure 3). We conclude that the elongate bones were deposited transverse to the predominant wave movement.

**Figure 4:**
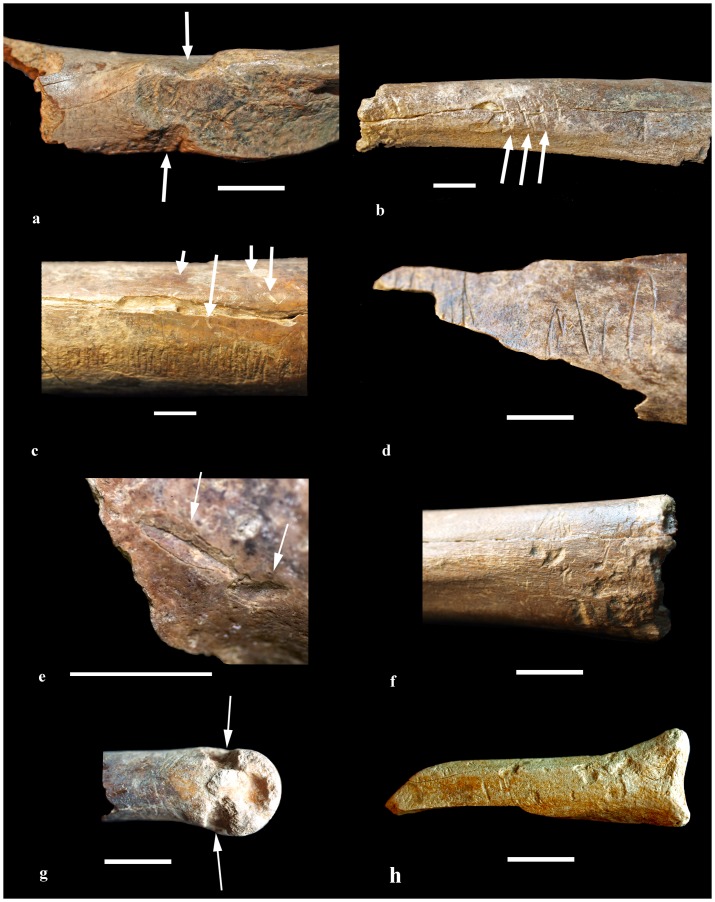
Bite marks on bones from the VVP skeletal deposits. (a) MV P231884 small *Macropus* ilium with paired v-shaped incisions cf. *Thylacoleo*; (b) MV P231885 *Macropus* 4^th^ metatarsal with v-shaped incisions cf. *Thylacoleo*; (c) MV P231886 distal *Macropus giganteus* tibial shaft with rodent gnawing, arrows indicate root etching; (d) MV P231887 *M. giganteus* tibial shaft with possible dasyurid bite marks; (e) MV P230123 macropodid limb fragment with depressed punctures cf. *Sarcophilus*; (f) MV P231888 chewed *Macropus* proximal 4^th^ metatarsal cf. *Sarcophilus*; (g) MV P230103 *Macropus* distal 4^th^ metatarsal with paired ?*Sarcophilus* canine or ?*Thylacoleo* incisor punctures; (h) MV P230090 small *Macropus* proximal 4^th^ metatarsal with bite marks and digestion damage cf. *Sarcophilus*. All scale bars equal 10 mm.

The lack of bone abrasion suggests that the bones had not been transported very far from where the animals had died and provides additional evidence that the bones are not reworked. Although the majority bones were dissociated, some associated *Macropus* foot bones were found in SA2. Of the total of 1028 bones and bone fragments collected, the presence of bite marks (Figure 4) on 7% of specimens indicates that animals fed on some of the carcasses prior to burial or submergence. This level of carnivore modification is similar to that documented by De Vis [38] (5%) and Price and Webb [39] (3–8%) for megafaunal deposits of a similar age in the Darling Downs in north-eastern Australia. The presence of greenstick fractures on many of the limb bones also indicates that the bones were broken through gnawing or trampling shortly after the animals' deaths. Root etching is present on 17.5% of specimens (Figure 4c), suggesting that plants growing on overlying sediments contributed to the diagenesis of the bones.

### Biting/gnawing traces

The majority of bones from the two deposits lack obvious bite marks, but those bite marks present are sufficiently distinctive to permit reasonably confident identification of the taxa responsible. Bite maker identifications can be made with a reasonable degree of certainty as the possible range of mammalian bite makers is limited to Tasmanian devils (*Sarcophilus*), thylacines (*Thylacinus*), the marsupial ‘lion’ (*Thylacoleo*), quolls (*Dasyurus*), marsupial mice (*Antechinus* or *Sminthopsis*) and rodents. The majority of these taxa have distinctive dental morphology and/or do not overlap in size range. The greatest potential for misidentification lies in distinguishing between thylacine and Tasmanian devil bite marks as they possess a similar dental morphology and the authors were unable to find any published analysis of the bite marks of the former.

There are four distinct types of trace marks left by teeth on skeletal fossils at the VVP site (Figure 4). These traces include: (i) many parallel grooves <1 mm wide, often paired with grooves on the other side of the bone (Figure 4c); (ii) limb bones with the ends often removed and many pits, scratches and depressed punctures 2–6 mm wide (Figure 4e–h); (iii) deep, straight, v-shaped grooves 3–7 mm wide and ∼20 mm long (Figure 4a–b); and (iv) non-parallel grooves ∼1 mm wide (Figure 4d). In addition, several bone fragments displayed surficial acid-etching consistent with the fragments having passed through an animal's gut (Figure 4h). Marks in group (i) are attributed to rodents and correspond to Category A of Sobbe [40] with the difference that, rather than one pair of incisors acting as an anchor point, in our sample both pairs of incisors (upper and lower) have left grooves. Previous excavations in the VVP area have also noted bite marks on bones attributed to rodents [35]. Group (ii) marks correspond to Categories D, G, I, 2 and 3 of Sobbe [40] and represent gnawing traces similar to those produced by Tasmanian devils or thylacines. Group (iii) marks correspond to Sobbe's [40] Category B and are attributed to *Thylacoleo carnifex*. Marks in group (iv) differ from those attributed to rodents in that the grooves are not straight and do not appear to have been caused by paired incisors. Marks in this group may represent grooves left by quoll (native cat) canines as they dragged across the bone surface. The vast majority of bite marks appear on appendicular elements of small macropodids, with very few wombat bones exhibiting bite marks despite their abundance at the site. Studies of *Sarcophilus* indicate that it is a generalist scavenger and will feed on any food source available [41]. When consuming the carcasses of larger prey it will often chew off the ends of long bones [42] or devour the carcass completely [40]. This feeding strategy matches the placement of some bite marks seen on specimens described in this study (Figure 4f–h). Studies of thylacine skull morphology indicate that it probably hunted small to medium bodied prey [43] and most of the bite-marked bones in this study fall into this size category. As such it is not possible to say for certain whether *Sacrcophilus*, *Thylacinus*, or a combination of both is responsible for the majority of bite marks on the skeletal fossils.

### Differences between records

**Figure 5:**
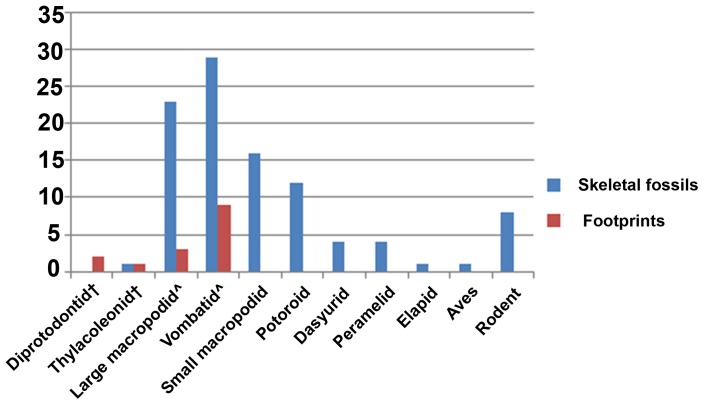
Relative abundance of taxa represented by trace and skeletal fossils. Graph displaying the number of individuals represented by trackways and body (skeletal) fossils at the VVP site. Taxa are arranged by approximate body mass, largest to smallest. † indicates extinct taxa, ∧ the taxa responsible for the macropodid and vombatid tracks at the site may be extinct.

**Figure 6:**
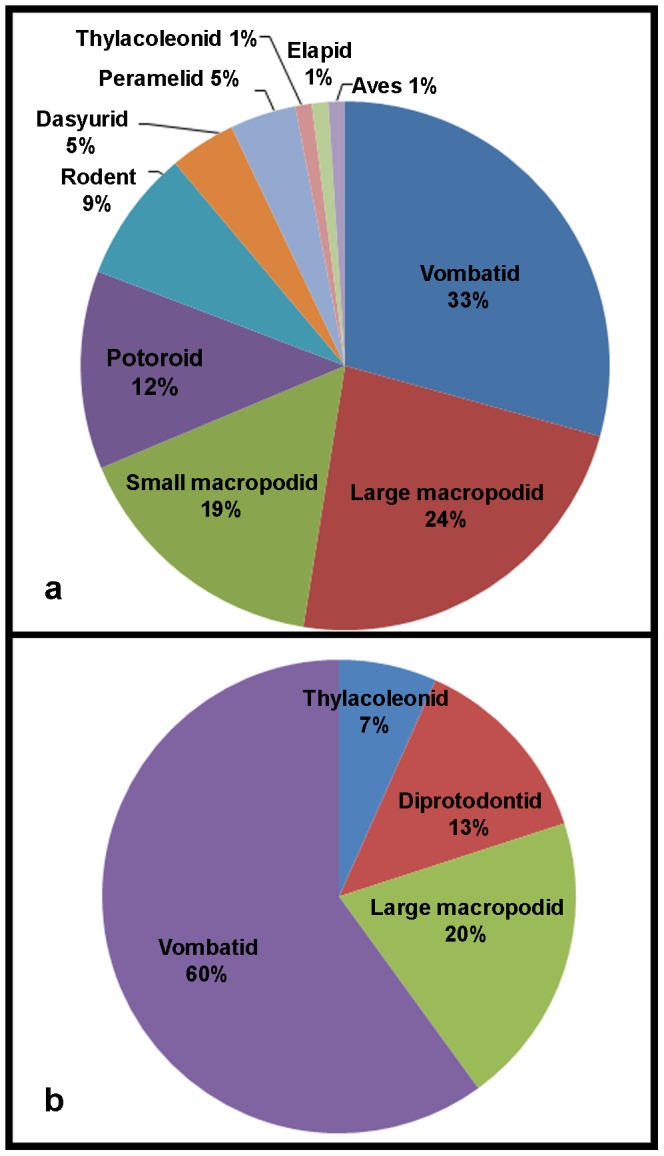
Proportional representation of taxa by trace and body fossils. Proportions of organisms represented by body (skeletal) fossils (a) and trace (footprint) fossils (b).

The body-fossil, footprint and bite-mark records (Figures 5, 6) differ in two ways: more individuals are represented by body fossils than by trace fossils; and the taxonomic composition of the three records differs. A list of all taxa present in the skeletal deposits can be found in Appendix S1.

Only larger taxa are represented by footprints, with smaller taxa such as wallabies and peramelids occurring only in the skeletal deposits and quolls and rodents occurring as both skeletal fossils and feeding traces (Table 2). Bite marks on bones provide a more complete record of the omnivorous/carnivorous component of the fauna than do the footprint and skeletal assemblages, with marsupial mice (e.g. *Antechinus* or *Sminthopsis*) possibly being the only mammalian candidates not represented. *Thylacoleo* is the only taxon at the site that appears to be represented by all three records.

**Table 2:** Taxa represented in the VVP fossil deposits.

Taxon	Body fossils (MNI)	Trackways	Bite marks
Large macropodid	23	3	-
Small macropodid	16	-	-
Potoroid	12	-	-
†Diprotodontid	-	2	-
Peramelid	4	-	-
Vombatid	29	9	-
†Thylacoleonid	1	1?	x
*Dasyurus*	4	-	x
*Thylacinus*	-	-	x
*Sarcophilus*	1	-	X
Rodent	8	-	X
Elapid	1	-	-
Aves	1	-	-

The number of individuals represented by body (skeletal) fossils, the number of trace fossils (trackways, after Carey et al. 2011) and the taxa possibly represented by bite marks on bones at the VVP site.

† indicates extinct taxa, x represents taxa whose bite marks are probably present and X represents taxa whose bite marks are confidently identified, MNI = minimum number of individuals.

There is a clear bias with respect to body size in the footprint and body fossil records present at the site: body fossils representing nearly the whole size range of the Pleistocene mammal fauna (except the largest), but the footprints representing only the larger taxa.

## Discussion

### Taphonomic setting

The low incidence of surficial weathering of the bones from SA1 and SA2 indicates relatively rapid burial or submergence. Most bones exhibit characteristics of weathering stage 0–1(after [44]), indicating that they probably lay on the surface for less than a year (assuming the VVP site had a similar climate to that in [44]). Given that Behrensmeyer [44] found that a third of the bones in an attritional assemblage (i.e. a bone assemblage deposited over an extended period of time) were likely to be “significantly weathered” (weathering stages 3–5), the relative lack of highly weathered bones in the VVP skeletal deposits suggests that the deposit accumulated over a short period. Alternatively, rapid transport of the bones to an aquatic environment, combined with permanent submergence and/or rapid burial, could explain the lack of advanced weathering. Submergence is indicated by the alignment of long bones transverse to the predominant wave direction (Figure 3). The high degree of bone fragmentation (no intact limb bones were recovered), and the presence of bite marks on several bones suggest that many of them derive from carcasses that were fed on by predators or scavengers. However, there is also evidence (in the form of associated fragments) that at least some of the fragmentation occurred *in situ*, suggesting that the bones may have been subject to post-depositional trampling or fragmentation by cracking-clays and root penetration.

Krapovickas et al. [28] described Miocene ichnocoenoses on “emergent sandy bars” of channel deposits and noted that the majority of footprints belonged to small vertebrates, with larger vertebrate footprints being comparatively scarce. Despite the depositional environment being somewhat similar, this is the opposite of what we observe at the VVP site, where small mammals are represented by body fossils but not footprints. The discrepancy is most likely due to differences in the substrates hosting the footprints.

### Comparison to the existing skeletal fossil record for the region

Skeletal fossil deposits are already known from a number of deposits in the lake beds of the VVP [34], [35], [36], [45]. Further afield, but still within southeastern Australia, the Late Pleistocene fauna has been exhaustively investigated through its occurrence in the Naracoorte Caves [45], [46] (approximately 200 km WNW of the VVP site). A list of the relevant taxa known from other late Pleistocene fossil sites in the region is given in Appendix S2. Taxa not previously reported from fossil sites in the VVP but found in this study include *Macropus* cf. *greyi*, *Wallabia bicolour*, *Lasiorhinus cf*, *krefftii* and the rodent, bird and elapid material. However, all of these taxa are know from the more complete late Pleistocene fossil records of southeastern South Australia [45], [46] and so this discrepancy probably reflects the small sample sizes associated with published deposits in the VVP.

### Body fossils versus trace fossils: paleoecological implications

One of the pitfalls of basing paleoecological inferences on ichnocoenoses is that there is no way to determine definitively the number of individuals responsible for the tracks; one organism can leave many trace fossils, but only one set of body fossils [2]. Although a single terrestrial vertebrate has the potential to leave many more trace fossils than body fossils, body fossils are less susceptible to weathering or erosion, and hence more likely to be preserved in the fossil record. In addition, skeletal fossils are easier to recognise and are thus more likely to be brought to the attention of paleontologists. Laporte and Behrensmeyer [30] noted that, on lake and river shores, the zone with potential for footprint preservation is only several tens of metres wide. This means that behavioral factors, such as whether an animal is foraging within this zone or just passing through, will have a large impact on the number of fossil prints recorded for a given taxon. The diprotodontid trackway at the VVP site indicates an individual moving from the shore into shallow water [12], suggesting that it was just passing through. However, in the case of the vombatid trackways, it is difficult to tell if there were many individuals present, or merely a few individuals moving around in the zone conducive to footprint preservation. Conversely, an estimate of the minimum number of individuals represented in a deposit of body fossils is easy to determine, but the deposit represents a time-averaged collection. Thus the fauna represented by body fossils also may fail to accurately reflect the population sizes of taxa in a given habitat at a specific moment in time.

Although it is often implied that taxa represented in body-fossil deposits all lived in the same habitat, the time-averaging effect seen in most of these deposits increases the likelihood that multiple habitats are sampled (due to vegetation change over time, or transportation of the body fossils after death). Western [47] discussed the problems with paleoecological inferences based on fossil assemblages. He suggested that, taphonomic bias aside, factors such as the lifespan, home range, population density, body size and preferred habitat of a taxon can have significant effects on its representation in a time-averaged skeletal fossil deposit. Western [47] also noted that the predator-to-prey ratio can have a significant effect on the survival of bones: the higher the ratio in the living population, the less likely bones are to survive. The footprints at the VVP site were most probably all formed in a single event spanning a few days or weeks [12], and so the trackways represent an actual faunal association. The skeletal fossils at the VVP site appear to have been deposited over a significantly longer period and thus tell a different story (Figure 5 and 6).

Lockley and Meyer [4] suggested that, in a deposit where both trace and body fossils occur, the presence of aquatic organisms may be indicated by skeletal fossils while terrestrial animals may instead be represented by trace fossils. Laporte and Behrensmeyer [30] also found, in Plio-Pleistocene deposits in Kenya, that aquatic vertebrate and invertebrate skeletal fossils were associated with terrestrial vertebrate trace fossils. At the VVP locality, all the body fossils and all the vertebrate trace fossils derive from terrestrial animals. Although this means that a narrower portion of the total faunal diversity is sampled (i.e. there are no aquatic vertebrates present), it provides a more complete picture of the terrestrial vertebrate fauna, as the complementary trace and skeletal fossil records help eliminate taphonomic bias.

### Implications for interpretation of Australian megafaunal sites

It is important to note that, although macropodids and vombatids were by far the most numerous taxa in the skeletal and trace fossil assemblages, the presence of *Thylacoleo* was detected through skeletal fossils, probable bite marks and a possible trackway. We suggest that a combination of behavioral and dietary factors increases the likelihood that large predators such as *Thylacoleo* be represented through trace fossils, both as bite marks on bones and as trackways. Another carnivore, *Thylacinus* (marsupial wolf), is possibly represented by bite marks but is absent from both the footprint and skeletal assemblages, possibly reflecting differences in hunting strategies and/or abundance at the site.

The largest taxon present at the VVP site, *Diprotodon optatum*, is absent from the skeletal deposits but represents the largest and most easily recognisable component of the trace fossil assemblage. In fact, it was the presence of the *Diprotodon* footprints that led to the initial discovery of the site, the presence of other trace fossils and the skeletal fossils only becoming apparent after closer inspection. *Diprotodon* is the most widespread and commonly recognised taxon in Australian skeletal megafaunal deposits, probably due to its size and the consequent ease with which its bones and teeth are retrieved and identified [48]. It is therefore suggestive of a taphonomic bias that skeletal fossils of *Diprotodon* are absent from the VVP site.

The factors affecting the proportional representation of various taxa comprising a fossil fauna vary greatly between skeletal and trace fossil assemblages. Western [47] noted that body size and death rate (average number of individuals to die in a given time period) were the two main factors affecting the number of individuals of a given taxon represented in a skeletal assemblage. The skeletons of smaller animals decompose more quickly than those of larger animals, but fewer skeletons of the latter group are deposited in a given time period due to their lower reproductive rates. Accordingly, it is the medium-sized animals (e.g. wildebeest and zebra in Amboseli National Park, Kenya) that are most commonly represented by skeletal remains [47]. A similar pattern is seen at the VVP site, with medium-to-large macropodids and vombatids making up the majority of the skeletal deposits (Figure 5a). Large-bodied taxa are likely to be over-represented in trace fossil assemblages for a number of reasons:

1. Larger footprints are easier to spot than small prints and are less likely to be mistaken for abiotic soft-sediment deformation;
2. Large footprints are less prone to erosion;
3. Preservation of large footprints is less dependent on substrate composition; and
4. Larger (heavier) animals are more likely to leave undertracks, meaning that their presence can be detected over a greater stratigraphic distance.

McNeil et al. [13] noted that without the presence of trace fossils, the existence of the two largest taxa at Wally's Beach (a late Pleistocene site in south-western Alberta, Canada), *Mammuthus* and *Camelops*, would be unknown. Similarly, despite *Diprotodon* being one of the most widely distributed and best known taxa in Australian late Pleistocene fossil deposits [48], it is absent from the skeletal fossil deposits at the VVP locality and is represented only by the fossil trackways. At the other end of the body-mass spectrum, rodent and small marsupial footprints are absent from the footprint record at the VVP site but their presence is clearly marked through the presence of skeletal fossils and bite marks on bones.

The Pleistocene fossil assemblage at the Victorian Volcanic Plains consists of footprints, skeletal fossils, and bite marks on the skeletal fossils. The two trace fossil records and the skeletal fossil record combine to allow a unique comparison of inherent bias in each of the three records. Our data suggest that trace fossils provide a more complete picture of the large-bodied faunal community and that skeletal fossils provide a better record of smaller-bodied taxa. The absence of the largest taxon from the skeletal fossil record is unexpected and highlights the fact that a faunal record derived from a skeletal deposit that accumulated over a significant time period can still be appreciably biased.

## Materials & Methods

The footprint assemblage from the Victorian Volcanic Plains (VVP) locality has been documented by Carey et al. [12]. It consists of 15 trackways (>700 tracks) created by three or four marsupial taxa (diprotodontid, macropodid, vombatid and possibly thylacoleonid). Specific ichnotaxonomic descriptions are outside the scope of this paper and are currently being prepared for publication elsewhere. Skeletal material was excavated from Skeletal Accumulations 1 and 2 (SA1 and 2) at the same locality using standard paleontological techniques. The lack of discrete sedimentological units within the deposits, combined with their shallowness (∼300 mm), meant that excavating in spits did not provide useful data. The locations of the excavated body fossils were recorded in relation to the grid used to plot the trace fossils. The orientations of a representative sample of limb bones (n = 48) and ripple marks (n = 336) were also measured in order to establish any sedimentary anisotropy of the fossils. Identification of the skeletal fossil material was undertaken at Museum Victoria (MV), where skeletal fossils and casts of the trace fossils from the VVP site are housed, and at the South Australian Museum (SAM). Although taxonomic identification to species level was possible for some specimens, the fragmentary nature of most specimens and the preponderance of postcranial material meant that in most cases specimens could only be identified to family or genus (see Appendix S3). In this study bones were classified into groups (e.g. large macropodid, small macropodid), rather than to species level, to facilitate comparison with the trackways (which could not be ascribed to track-makers at the species level in most cases [12]). Body mass estimates were taken from Wroe et al. [49], [50], [51] and Strahan and van Dyck [52]. Exact site coordinates are not given due to the sensitive nature of the site. The authors should be contacted for further detail relating to the site location. No specific permits were required for the described field studies.

## Supporting Information

Appendix S1 **Faunal list for the VVP skeletal deposits.** A list of all identified taxa represented in the skeletal fossil deposits at the Victorian Volcanic Plains site.
(DOCX)

Appendix S2 **List of late Pleistocene vertebrate taxa recorded by various authors for south-eastern Australia.**
(DOCX)

Appendix S3 **List of the specimens catalogued from the VVP site (now housed in the MV collection).**
(XLSX)
