# Supplementary material for: Contemporaneous Trace and Body Fossils from a Late Pleistocene Lakebed in Victoria, Australia, Allow Assessment of Bias in the Fossil Record
Source: PLoS One. 2013 Jan 2;8(1):e52957. doi: 10.1371/journal.pone.0052957 (PMC3534647; doi:10.1371/journal.pone.0052957)
Supplement: Appendix S1 — Faunal list for the VVP skeletal deposits. A list of all identified taxa represented in the skeletal fossil deposits at the Victorian Volcanic Plains site. (DOCX) [file pone.0052957.s001.docx]

APPENDIX 1. Taxa recorded from Skeletal Accumulation 1 and 2 at the VVP locality.

Gastropoda

Pulmonata

Reptilia

Squamata

Elapidae

Aves

Mammalia

Marsupialia

Dasyuromorphia

Dasyuridae

*Sarcophilus* cf. *harrisii*

*Dasyurus* cf. *viverrinus*

Peramelemorphia

Peramelidae

cf. *Perameles*

Diprotodontia

Phalangerida

Potoroidae

cf. *Bettongia*

Macropodidae

*Macropus giganteus*

*M.* cf. *ferragus*

*M. rufogriseus*

*M.* cf. *greyi*

*Wallabia bicolor*

*†Protemnodon* sp.

Vombatiformes

Vombatidae

*Lasiorhinus* sp.

†*Lasiorhinus* cf. *krefftii*

Thylacoleonidae

†*Thylacoleo carnifex*

Placentalia

Rodentia

Muridae

*Rattus* sp.
