# Supplementary material for: Contemporaneous Trace and Body Fossils from a Late Pleistocene Lakebed in Victoria, Australia, Allow Assessment of Bias in the Fossil Record
Source: PLoS One. 2013 Jan 2;8(1):e52957. doi: 10.1371/journal.pone.0052957 (PMC3534647; doi:10.1371/journal.pone.0052957)
Supplement: Appendix S2 — List of late Pleistocene vertebrate taxa recorded by various authors for south-eastern Australia. (DOCX) [file pone.0052957.s002.docx]

**Murray 1991 p. 1127** [1]

Megafaunal species in the south-eastern region of Australia

Marsupialia

Dasuridae

*Sarcophilus* spp.

Phascolarctidae

*Phascolarctos cinereus*/*stirtoni*

Diprotodontidae

*Diprotodon optatum*

*Zygomaturus trilobus*

Palorchestidae

*Palorchestes azael*

Thylacoleonidae

*Thylacoleo carnifex*

Macropodidae

*Propleopus oscillans*

*Procoptodon rapha*

*Sthenurus gilli*

*Sthenurus atlas*

*Sthenurus maddocki*

*Sthenurus andersoni*

*Simosthenurus occidentalis*

*Protemnodon roechus*

*Macropus ferragus*

Monotremata

*Megalibgwilia ramsayi*

Aves

*Progura naracoortensis*

Reptilia

*Wonambi naracoortensis*

**Reed and Bourne 2000** [2]

Selected species known from late Pleistocene deposits in south-eastern South Australia

Marsupialia

Dasyuridae

*Sarcophilus laniarus*

*Dasyurus geoffroii*

*Dasyurus maculatus*

*Dasyurus viverrinus*

Thylacinidae

*Thylacinus cynocephalus*

Peramelidae

*Isoodon obesulus*

*Perameles bougainville*

*Perameles gunnii*

Phascolarctidae

*Phascolarctos stirtoni*

Diprotodontidae

*Zygomaturus trilobus*

*Diprotodon optatum*

Palorchestidae

*Palorchestes azael*

*Palorchestes parvus*

Vombatidae

*Lasiorhinus krefftii*

*Warendja wakefieldi*

Thylacoleonidae

*Thylacoleo carnifex*

Macropodidae

*Propleopus oscillans*

*Procoptodon goliah*

*Procoptodon rapha*

*Procoptodon browneorum*

*Procoptodon gilli*

*Simosthenurus baileyi*

*Simosthenurus maddocki*

*Metasthenurus newtonae*

*Simosthenurus occidentalis*

*Simosthenurus pales*

*Sthenurus andersoni*

*Congruus congruus*

*Lagorchestes leporides*

*Lagorchestes fasciatus*

*Macropus eugenii*

*Macropus fuliginosus*

*Macropus giganteus titan*

*Macropus greyi*

*Macropus rufogriseus*

*Onychogalea lunata*

*Protemnodon anak*

*Protemnodon brehus*

*Protemnodon roechus*

*Wallabia bocolor*

Monotremata

*Megalibgwilia ramsayi*

Placentalia

Rodentia

*Rattus fuscipes*

*Rattus lutreolus*

*Rattus tunneyi*

Numerous small birds and reptiles

**Roberts et al. 2001** [3]

Extinct megafaunal species in the VVP lakes region of Australia

Maruspialia

Dasyuridae

*Sarcophilus laniarus*

Diprotodontidae

*Diprotodon optatum*

*Diprotodon* sp.

*Zygomaturus* sp.

Vombatidae

*Phascolonus gigas*

Thylacoleonidae

*Thylacoleo carnifex*

Macropodidae

*Procoptodon* sp.

*Sthenurus* sp.

*Simosthenurus* sp.

*Protemnodon anak*

*Protemnodon cf. brehus*

*Macropus giganteus titan*

**De Vis 1899** [4]

Extinct megafaunal species in Lake Colongulac

Marsupialia

Macropodidae

*Procoptodon goliah*

*Macropus* (*pan*) *cf. ferragus*

**Marshall 1974** [5]

Taxa known from late Pleistocene deposits from Keilor (Dry Creek LF)

Marsupialia

Dasyuridae

*Sarcophilus laniarus*

Thylacinidae

*Thylacinus cynocephalus*

Peramelidae

*Perameles nasuta*

Diprotodontidae

*Zygomaturus trilobus*

Vombatidae

*Vombatus ursinus*

Thylacoleonidae

*Thylacoleo carnifex*

Macropodidae

*Protemnodon anak*

*Protemnodon brehus*

*Macropus giganteus titan*

*Macropus cf. ferragus*

*Macropus agilis*

*Macropus rufogriseus*

**Marshall 1974** [5]

Taxa known from late Pleistocene deposits from Lake Colongulac

Marsupialia

Dasyuridae

*Sarcophilus laniarus*

Thylacinidae

*Thylacinus cynocephalus* (*rostralis*)

Diprotodontidae

*Diprotodon optatum*

Vombatidae

*Lasiorhinus* sp.

*Vombatus ursinus*

Thylacoleonidae

*Thylacoleo carnifex*

Macropodidae

*Procoptodon rapha*

*Sthenurus* sp.

*Protemnodon anak*

*Protemnodon brehus*

*Macropus rufogriseus*

*Macropus agilis cf. siva*

*Magcropus giganteus titan*

*Thylogale billardierii*

**Errey and Flannery 1978** [6]

Taxa known from late Pleistocene deposits from Lake Colongulac

Maruspialia

Dasyuridae

*Sarcophilus laniarus*

Thylacinidae

*Thylacinus cynocephalus* (*rostralis*)

Vombatidae

*Lasiorhinus* sp.

Thylacoleonidae

*Thylacoleo carnifex*

Diprotodontidae

*Diprotodon* sp.

*Zygomaturus* sp.

Macropodidae

*Procoptodon* sp.

*Simosthenurus* sp.

*Protemnodon brehus* or *roechus*

*Protemnodon anak*

*Macropus giganteus titan*

*Aepyprymnus*

1. Murray PF (1991) The Pleistocene megafauna of Australia. In: Vickers-Rich P, Monaghan JM, Baird RF and Rich TH, editors. Vertebrate Palaeontology of Australasia: Pioneer Design Studio. pp. 1071-1164.

2. Reed EH, Bourne SJ (2000) Pleistocene fossil vertebrate sites of the south east region of South Australia. Transactions of the Royal Society of South Australia 124: 61-90.

3. Roberts RG, Flannery TF, Ayliffe LK, Yoshida H, Olley JM, Prideaux GJ, Laslett GM, Baynes A, Smith MA, Jones R, Smith BL (2001) New Ages for the last Australian megafauna: Continent-wide extinction about 46,000 years ago. Science 292: 1888-1892.

4. De Vis CW (1899) On some remains of marsupials from Lake Colongulac, Victoria. Proceedings of the Royal Society of Victoria 12: 107-111.

5. Marshall LG (1974) Late Pleistocene mammals from the "Keilor Cranium Site", southwestern Victoria, Australia. Memoirs of the National Museum of Victoria 13: 63-85.

6. Errey K, Flannery TF (1978) The neglected megafaunal sites of the Colongulac region, western Victoria. The Artefact 3: 101-106.
